# A curated database reveals trends in single-cell transcriptomics

**DOI:** 10.1093/database/baaa073

**Published:** 2020-11-28

**Authors:** Valentine Svensson, Eduardo da Veiga Beltrame, Lior Pachter

**Affiliations:** Division of Biology and Biological Engineering, California Institute of Technology, 1200 E California Blvd, Pasadena, CA, 91125, USA; Division of Biology and Biological Engineering, California Institute of Technology, 1200 E California Blvd, Pasadena, CA, 91125, USA; Division of Biology and Biological Engineering, California Institute of Technology, 1200 E California Blvd, Pasadena, CA, 91125, USA

## Abstract

The more than 1000 single-cell transcriptomics studies that have been published to date constitute a valuable and vast resource for biological discovery. While various ‘atlas’ projects have collated some of the associated datasets, most questions related to specific tissue types, species or other attributes of studies require identifying papers through manual and challenging literature search. To facilitate discovery with published single-cell transcriptomics data, we have assembled a near exhaustive, manually curated database of single-cell transcriptomics studies with key information: descriptions of the type of data and technologies used, along with descriptors of the biological systems studied. Additionally, the database contains summarized information about analysis in the papers, allowing for analysis of trends in the field. As an example, we show that the number of cell types identified in scRNA-seq studies is proportional to the number of cells analysed.

**Database URL**: www.nxn.se/single-cell-studies/gui

## Introduction

The availability of large numbers of comprehensive single-cell transcriptomics studies (16) is making possible the study of biological variation in unprecedented detail (5). One interesting aspect of this ‘big data’ biology consisting of a large set of measurements from many cells is that it can yield insights even after initial published analysis of individual datasets. Moreover, with hundreds of datasets available, integration becomes a powerful tool for exploration. However, integration of diverse datasets requires standardization in how data is collected, shared and curated (15).

A number of ‘atlas’ projects have been launched to address this problem and to assist researchers in focused domains. For example, The ‘Human Cell Atlas’ portal aims to provide uniformly processed single-cell genomics data from all of the human body (12). ‘JingleBells’ provides single-cell data, with a focus on immune cells (11). The ‘conquer’ database provides uniformly processed single-cell expression data to facilitate benchmarking of computational tools (14). The ‘PanglaoDB’ database provides single-cell RNA-seq count matrices from public sequencing data in the National Center for Biotechnology Information Sequence Read Archive (3). The ‘EMBL-EBI Single-Cell Expression Atlas’ provides uniformly processed data from submissions to ‘ArrayExpress’ (https://www.ebi.ac.uk/gxa/sc/home). The Broad Institute offers a ‘Single-Cell Portal’ which can be used to share custom scRNA-seq data (https://portals.broadinstitute.org/single_cell). A database called ‘scRNASeqDB’ provides links to a number of datasets from human scRNA-seq experiments (1). These efforts all aim to tackle different aspects of the considerable challenge of data management resulting from the extraordinary rapid adoption of single-cell genomics technologies.

We focus on a missing resource, namely a database of single-cell transcriptomics studies generating data and results, rather than primary data. The compilation of such a database required us to read and manually curate large numbers of publications, which we indexed according to publication and study authors. Our database will allow researchers interested in specific tissues to rapidly identify relevant studies. Furthermore, by virtue of providing a comprehensive overview of the field, our database can highlight understudied tissues. The database will facilitate appropriate citation of previous work when performing follow-up experiments. This database tracks metadata applicable to most studies, such as the number of cell types identified, and protocols used. We show that these annotations enable analysis of trends in the field.

## Database structure

This database aims to provide a link between datasets from different tissues, pointers to data location and relevant references. Together, these attributes make published data and results readily discoverable. A secondary goal is to annotate useful metadata associated with the primary studies.

The ‘Single-cell studies database’ considers the analysis of many genes at once in single cells as a ‘single-cell transcriptomics’ study. To allow for comprehensive coverage within a meaningful domain, the scope of the database was restricted in certain ways. For example, multicolor fluorescence flow cytometry and mass cytometry experiments were not included, even though both technologies can measure dozens of analytes per cell. The focus was restricted to datasets with the expression of more than a hundred genes measured in individual cells. Some targeted technologies measuring fewer genes such as osmFISH were also included when they could be directly related to higher throughput counterparts (2, 13, 19).

In studies performing multi-omics experiments, only cells with transcriptome readout are considered. This does include data where multiple omics are measured in the same cells, such as simultaneous RNA and DNA sequencing (7).

The primary identifier of each entry in the database is the canonical digital object identifier (DOI) of a publication. Based on the DOI, four entries are included using the CrossRef API (https://github.com/CrossRef/rest-api-doc): ‘Authors’, ‘Journal’, ‘Title’ and ‘Date’. Additional fields are based on the contents of the publication and are manually annotated by investigating the text and supplementary material of the publication. If the study was deposited to the bioRxiv (https://www.biorxiv.org), the ‘bioRxiv DOI’ field indicates this. Attributes include:

- Reported cells total: the number of cells investigated in the study.
- Technique: the technology or protocol used.
- Panel size: the number of genes investigated when targeted technologies such as multiplexed smFISH were applied.
- Measurement: the type of quantitative measurements performed (e.g. RNA-seq, *in Situ* or microarray).
- Data location: the public repository accession ID for the raw data.
- Organism: the species of origin of cells examined in the study.
- Tissue: the tissue type from which single cells were collected.
- Cell source: notes about the cells in the study.
- Disease: indicates disease states for which cells were investigated in the study.
- Contrasts: the different experimental conditions studied, if any.
- Isolation: the method used to produce the single-cell suspension.
- Developmental stage: the developmental stages or ages of the organism’s cells were collected from.

Additionally, some fields are binary corresponding to a ‘Yes’ or ‘No’ entry. This is used for the following attributes:

- Cell clustering:  did the study perform unsupervised clustering of cells (4)?
- Pseudotime: were cellular trajectories inferred with pseudotime methods (9)?
- RNA velocity: was a vector field inferred from spliced and unspliced reads (6)?
- PCA: was a principal component analysis performed?
- tSNE:  was the t-Distributed Stochastic Neighbor Embedding algorithm used for visualization (18)?

Finally, the number of cell types or clusters identified in each study is recorded under Number of reported cell types or clusters. This is most commonly based on *de novo* clustering, but in some cases, it is based on the number of distinct presorted cell types.

Fields may be added over time as new forms of data annotation and analysis become popular over time. Addition of new fields to existing studies is a quicker process than starting without a list of studies.

While the manual curation of the data made possible description of numerous details from the papers in the database, some entries are missing due to difficulty in finding information. However, we believe the overall content of the database is substantial enough to serve as a good starting point for the community to contribute and fill in the gaps. We show that even with some missing annotation, the database in its current form makes possible analysis of trends in the field.

The database can be accessed via a graphical user interface (GUI) using Google Sheets at http://www.nxn.se/single-cell-studies/gui. This view allows searching on keywords and for browsing studies. One tab, ‘Count summaries’, lists all unique tissues, techniques, measurements and diseases present in the database with the number of studies they appear in. Importantly, this GUI also allows for the contribution of information to the database through comments on individual entries.

A version of the database in tab-separated values (TSV) format can be downloaded from www.nxn.se/single-cell-studies/data.tsv. This enables researchers to perform analyses using the data.

New studies can be submitted through a form located at http://www.nxn.se/single-cell-studies/submit. Submissions require a DOI. The form also allows for entry of additional metadata through optional fields. Claims in the submissions are spot checked to ensure they refer to the original text in the publication. Each new entry or change to the database is performed manually on a case-per-case basis.

Corrections to existing entries are also performed individually on a case-per-case basis as curators become aware of mistakes. Users can make curators aware of mistakes by creating a new submission with the existing DOI and mention the mistake in the free text ‘Other notes’ field. Additionally, any user can comment on individual cells in the Google Sheets GUI or make direct contact with curators.

Every day a snapshot of the database is saved (in TSV format) using Google Cloud Functions, and all these snapshots are available in a public Google Storage bucket at gs://single-cell-studies. An example snapshot is provided as **Supplementary Table 1**, which has data on 550 studies published between 2003 and August 17, 2019.

## Results

**Figure 1.**
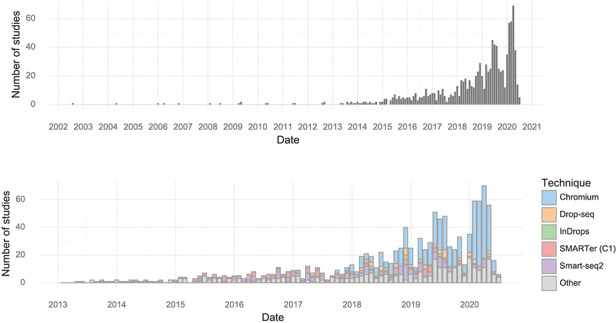
Studies over time. (Upper) The number of single-cell transcriptomics studies published per month. (Lower) The number of scRNA-seq studies published per month stratified by method.

The earliest single-cell transcriptomics study recorded in the database was published in 2004. Since 2013, almost every month at least one study has been published. The rate of study publication has increased steadily, and in January, February and March of 2020, there were over 50 single-cell transcriptomics studies published per month (Figure 1). In the first half of 2020, the median scRNA-seq study investigated approximately 31,000 cells (Table 1).

**Table 1.** Single-cell study trends (left) Number and size of single-cell transcriptomics studies in 2019. (middle) Most common tissue investigated with single-cell transcriptomics. (‘Culture’ refers to in vitro studies of cell lines). (right) Journals which have published most single-cell transcriptomics studies. (‘bioRxiv’ means the study is so far only available on bioRxiv).

	Monthly statistics			Top tissues		Top journals	
	Month	Studies	Median cells	Tissue	Studies	Journal	Studies
**0**	November 2019	12	39,895	Brain	171	bioRxiv	136
1	December 2019	29	15,601	Culture	107	Nature	82
2	January 2020	57	35,173	Blood	35	Cell	73
3	February 2020	50	36,044	Pancreas	33	Nat Commun	70
4	March 2020	68	31,514	Lung	32	Cell Reports	66
5	April 2020	38	30,396	Heart	25	Science	48
6	May 2020	13	22,000	Bone marrow	22	Cell Stem Cell	28

**Figure 2.**
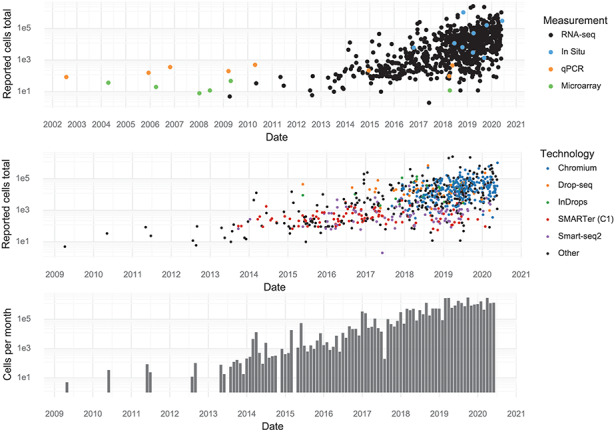
Scale of experiments and data over time. (Upper): The number of cells measured in a study, stratified by the measurement method. (Middle): The number of cells measured in scRNA-seq experiments, stratified by scRNA-seq protocol. (Lower): The aggregate number of cells measured per month.

Individual studies have increased in scale over time, and every few months, a new study is released that breaks the previous record in terms of number of cells assayed. During the first half of 2020, approximately 1,400,000 cells were added to the pool of public data every month (Figure 2).

**Figure 3.**
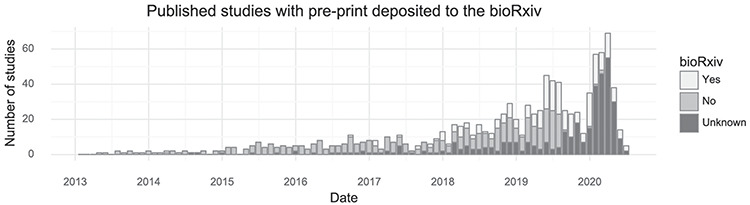
Preprint usage over time. The number of studies published in a given month stratified by whether they at some point were deposited to bioRxiv. (Including studies currently only available on bioRxiv).

Many tissues have been investigated by single-cell transcriptomics methods, but the brain is the most popular with 171 associated citations out of 1033 (Table 1). Another trend observed from this database is that authors of single-cell transcriptomics papers are increasingly making use of the bioRxiv preprint server. In total, 254 of 1033 studies were deposited to bioRxiv (25%, Figure 3). Single-cell studies are published in many different journals, with *Nature* and *Cell* having published the most (Table 1). The increasing popularity of these kinds of studies means the field, as measured by the number of active authors, has grown over time. Since 2016, the cumulative number of unique authors has doubled approximately every 15 months (**Supplementary Figure 1**).

Additionally, we used the Open Citations API (https://opencitations.net/) to obtain cross-citation numbers between all articles in the database. Studies on brain (6921 citations total) and cell cultures (6026 citations total) had the most citations (Table 2a). The most cited studies in the database were Macosko *et al*. (8) (190 citations out of 1036) and Trapnell *et al*. (17) (124 citations out of 1036, Table 2b).

**Table 2.** Top citation summary, a) Top cited tissues. b) Single-cell studies most cited by other single-cell studies.

DOI	Citations	Shorthand	Date
10.1016/j.cell.2015.05.002	190	Macosco et al. Cell	2015-05
10.1038/nbt.2859	124	Trapnell et al. NBT	2014-03
10.1038/nbt.3192	114	Satija et al. NBT	2015-04
10.1038/ncomms14049	107	Zheng et al. NComm	2017-01
10.1016/j.cell.2015.04.044	77	Klein et al. Cell	2015-05
10.1038/nmeth.2639	72	Picelli et al. NMeth	2013-09
10.1126/science.aad0501	72	Tirosh et al. Science	2016-04
10.1126/science.aaa1934	57	Zeisel et al.	2015-02
10.1038/nature12172	54	Shalek et al. Nature	2013-05
10.1038/nmeth.2645	54	Brennecke et al. NMeth	2013-09

**Figure 4.**
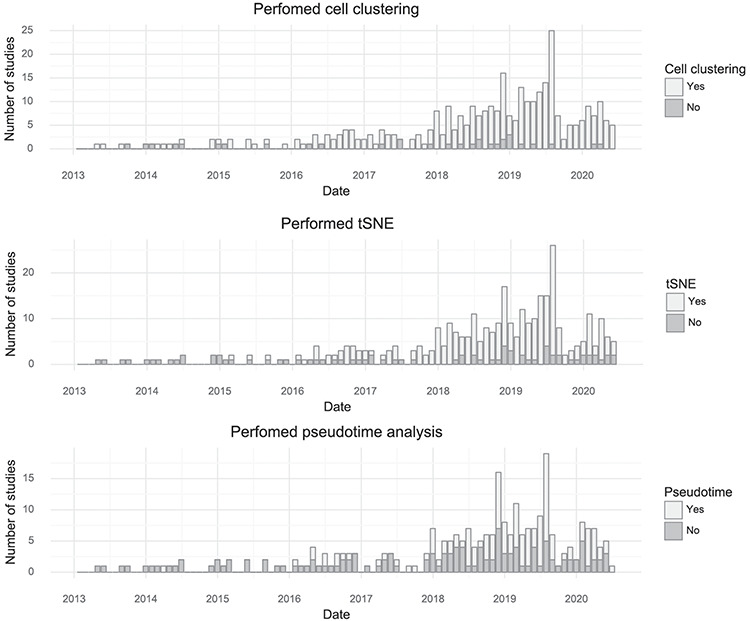
Popularity of forms of analysis over time. (Top) The number of studies doing clustering per month. (Middle) The number of studies using t-SNE per month. (Bottom) The number of studies doing pseudotime analysis per month.

By tracking the types of analyses performed with single-cell transcriptomics data, it is possible to learn something about what the community as a whole is aiming to learn from the assays. The most common application is to survey molecular ‘cell types’ by clustering cells based on gene expression. Almost every study performs clustering (90%). The t-SNE visualization method became nearly universally applied after its first use for single-cell analysis in 2015, although the fraction of studies per month using it has decreased slightly in the last year, possibly due to the introduction of Uniform Manifold Approximation and Projection (10). ‘Pseudotime’ is less frequently examined but is still very popular with about half of published studies investigating pseudotime trajectories (Figure 4).

**Figure 5.**
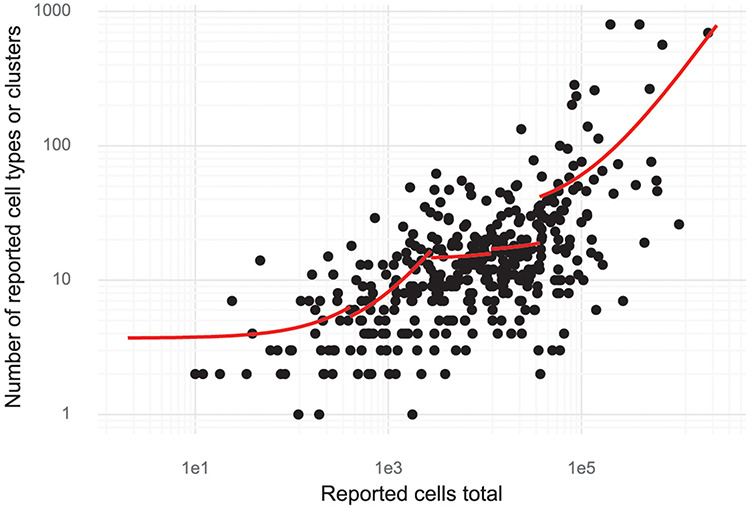
Cluster and cell numbers. The number of cells studied versus the number of clusters or cell types reported in a study. Red curves correspond to linear regression stratified to five quantiles of ‘Reported cells total’.

Since *de novo* clustering and cell type discovery is almost always performed, we annotated the number of clusters of cells identified in the studies. This revealed a high correlation between cell type numbers and the number of cells investigated. For small studies (up to about 1000 cells), on average, one cell type is identified per 150–200 cells assayed (linear regression, see ‘**Methods**’). For large studies with hundreds of thousands of cells, the rate is closer to one cell type per 3000 cells assayed (Figure 5). Stratifying the studies by the 11 most studied tissues showed that most tissues have a significant dependency between cells studied and cell types or clusters (log-linear regression, Wald test). The exceptions were blood, bone marrow, skin and kidney (**Supplementary Figure 2**).

## Discussion

The curated database described here is hosted at https://www.nxn.se/single-cell-studies. It has been designed for easy access to the underlying data and for in depth analysis in Python or R. The database was designed to facilitate access to published single-cell research, so that, for example, a researcher can find all single-cell studies of the pancreas to explore the results and analyse public data. We found that analysis of other aspects of the studies described in the papers, namely attributes such as type of protocol, number of cells or the number of clusters identified, revealed interesting trends in the field. We believe that our finding that the number of clusters identified is directly proportional to the number of cells analysed merits some scrutiny in light of the biological significance that is frequently associated with the number of clusters detected.

The database is also designed to enable contributions by the community via a mechanism for suggesting additions, adding data and for commenting. Forms for these functions are hosted at https://www.nxn.se/single-cell-studies/gui and https://www.nxn.se/single-cell-studies/submit.

## Methods

The growth rate of single-cell transcriptomics authorships was studied by taking the cumulative unique new authors each month, then calculating the month-to-month percent increase. The doubling time in months was calculated as log(2)/log(1 + [yearly average monthly change]).

To investigate the relation between the number of reported cell types of clusters and the reported cells total we divided the data into five quantiles based on the log10(reported cells total). For each quantile, we fitted a linear regression [cell types] = b * cells + c, so that 1/b corresponds to the number of cells needed to increase the number of cell types by 1.

For tissue-specific trends in reported cells versus number of clusters, we identified the 11 most studied tissues. For each tissue, we fitted a linear regression log([cell types]) = b * log(cells) + c and evaluated the Wald test *P*-value for the coefficient b. The relation between number of cells and number of clusters was determined to exist if the *P*-value was less than 0.05.

## Supplementary Material

baaa073_Supp
