# Supplementary figures and images for: A curated database reveals trends in single-cell transcriptomics
Source: Database (Oxford). 2020 Nov 28;2020:baaa073. doi: 10.1093/database/baaa073 (PMC7698659; doi:10.1093/database/baaa073)

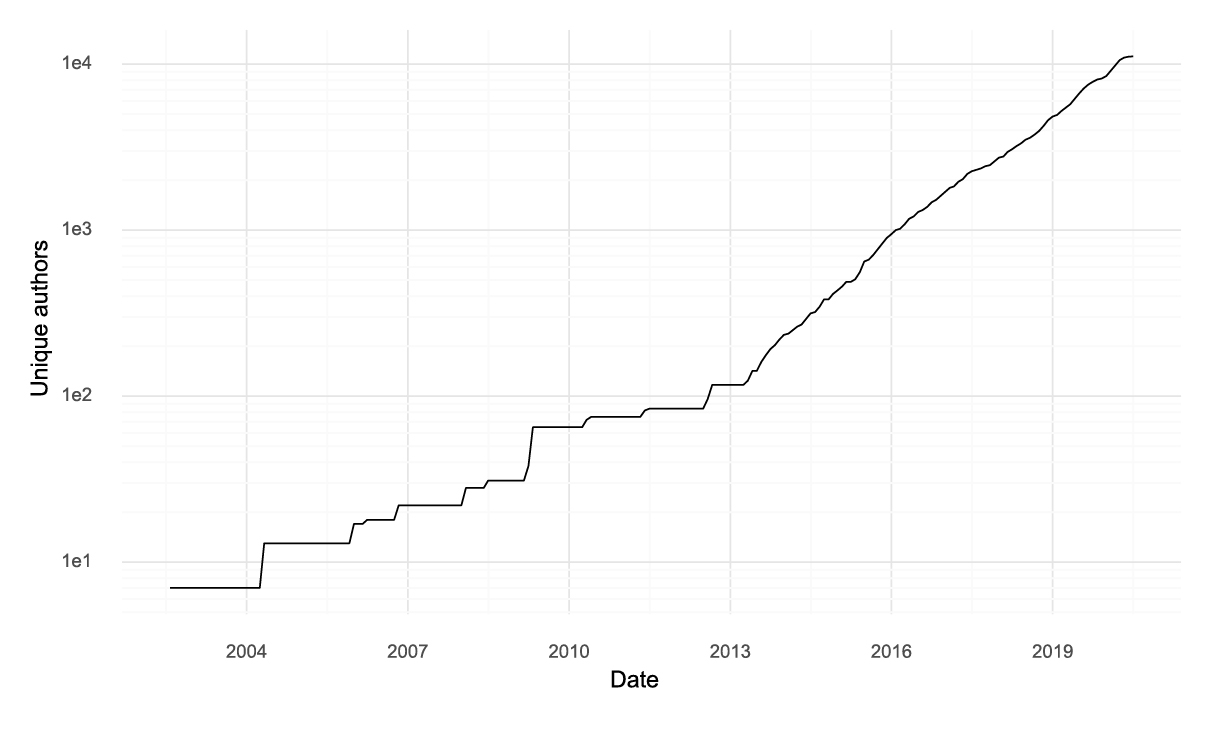

Supplement: baaa073_Supp [file baaa073_supp.zip › Supp. Fig. 1@4x-100.jpg]

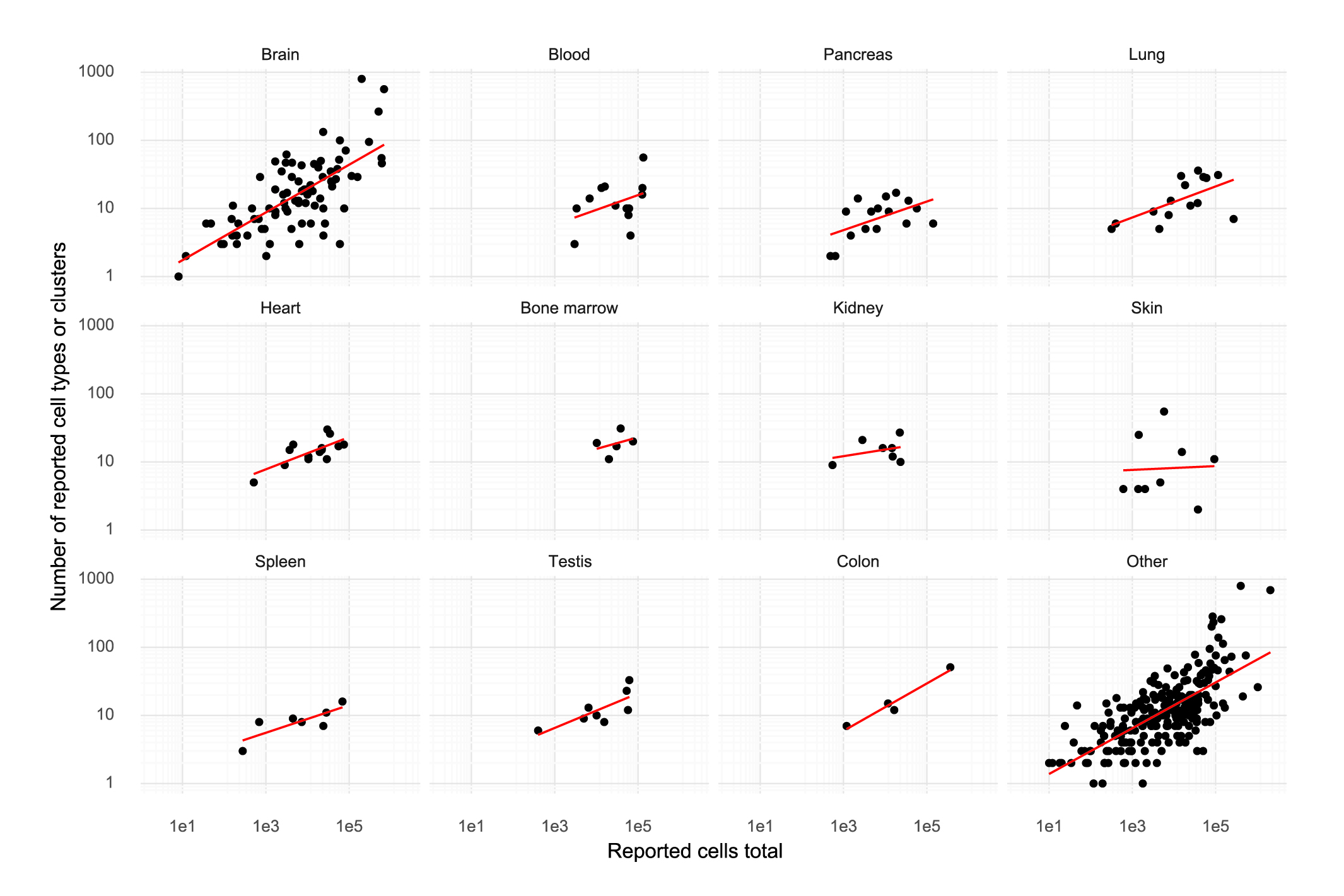

Supplement: baaa073_Supp [file baaa073_supp.zip › Supp. Fig. 2@4x-100.jpg]
